# Indian teachers’ and parents’ perceptions and experiences of food and nutrition education in primary schools: mixed-method study

**DOI:** 10.3389/fnut.2025.1635389

**Published:** 2025-08-06

**Authors:** A. Wincie Wilmah, R. K. Jaishree Karthiga

**Affiliations:** School of Social Sciences & Languages, Vellore Institute of Technology, Chennai, India

**Keywords:** food and nutrition education (FNE), sustainable development goals, primary schools, nutritional education, beneficial diet plans, mixed-method study

## Abstract

**Introduction:**

Food and nutrition education (FNE) is vital for shaping lifelong dietary behaviours and advancing sustainable development goals related to food security and responsible consumption. Despite global initiatives, India lacks a standardized framework for FNE in primary schools, resulting in inconsistent curricula and limited teacher preparedness.

**Methodology:**

This mixed-method study examined 350 stakeholders, including 110 primary school teachers, 187 parents, and 53 students in Tamil Nadu through structured surveys and qualitative interviews. Quantitative findings revealed that 73% of participants supported weekly FNE sessions.

**Results:**

76% of teachers reported inadequate training in food processing and nutrition, and 68% preferred FNE as a standalone subject. Statistically significant differences emerged between urban and rural stakeholders in sustainability awareness (*χ*^2^ = 10.94, *p* < 0.01), and teacher confidence in nutrition education correlated positively with successful FNE implementation (*r* = 0.68, *p* < 0.001).

**Analysis:**

Qualitative analysis identified three key themes: (1) limited institutional frameworks for sustainable FNE, (2) strong sociocultural influences on food choices, and (3) disparities in sustainability awareness across regions.

**Discussion:**

The findings support policy-driven reforms, such as mandatory teacher training, curriculum restructuring to embed FNE, and community engagement programmes. This study contributes practical insights for education policymakers aiming to enhance children’s food and nutrition literacy and promote sustainable dietary practices across Indian primary schools.

## Introduction

Food and nutrition education (FNE) plays a critical role in fostering healthy eating behaviours, promoting food and nutrition literacy, and supporting sustainable development goals (SDGs) related to food security and wellbeing. FNE has emerged as an essential component of school curricula worldwide, especially in the wake of global health crises and shifting sustainability imperatives. International frameworks such as UNESCO’s 2024/25 Global Education Monitoring Report and the OECD’s “Trends Shaping Education 2025” emphasize the urgent need to embed nutrition and food literacy into formal education systems, acknowledging its role in fostering lifelong health, resilience, and environmental stewardship. In India, national efforts under the NEP 2020 and flagship programs like PM POSHAN and Samagra Shiksha underscore a policy-level commitment to integrating health, nutrition, and wellbeing in early childhood and primary education. However, despite these endorsements, FNE often remains under-implemented or unevenly embedded in classroom practice, especially in resource-constrained settings.

Although FNE has gained policy-level visibility in India through initiatives like PM POSHAN and the National Education Policy (NEP) 2020, there remains a critical gap in the literature that examines how primary school teachers interpret and implement FNE within classroom settings. Existing research largely discusses broader themes, such as educational reform (1) and teacher identity (2); yet, there is a noticeable absence of subject-specific inquiry into educators’ pedagogical agency regarding FNE. Tambat (3) further emphasized the systemic oversight in teacher education concerning nutrition-related instruction, reinforcing the need for empirical studies that center teachers’ voices in curriculum integration. This study addresses that lacuna by foregrounding Indian teachers’ experiences as an essential lens for understanding FNE implementation at the grassroots level. This study seeks to address this gap by exploring structured, context-sensitive approaches to FNE, particularly through interdisciplinary and feminist pedagogical lenses.

While several countries have integrated FNE into primary education through structured curricula, its integration in Indian primary schools remains underdeveloped, often informal, and inconsistently applied. Existing research on FNE has predominantly focused on nutritional policies, dietary habits, and school-based intervention programmes, both globally and within India. International studies have explored barriers to FNE implementation from the perspective of educators, such as in Australia, where teachers reported challenges related to resources and curriculum support (4). However, within the Indian context, there remains a notable gap in the literature that systematically examines primary school teachers’ perspectives on FNE as a pedagogical priority. While national policies like PM POSHAN and Samagra Shiksha promote nutrition-related initiatives, empirical studies capturing educators’ lived experiences and instructional agency in integrating FNE into curricula are limited. This study addresses that gap by foregrounding Indian teachers’ voices as a critical lens for understanding effective curriculum integration.

### Background of the study

In India, FNE has historically been addressed through fragmented programmes led by ministries, such as Health and Family Welfare, Education, and Women and Child Development. The Integrated Child Development Services (ICDS), Midday Meal Scheme, and School Health Program have implicitly included elements of nutrition awareness, but none have formalized FNE as a pedagogical subject within the primary education curriculum. NEP 2020, while promoting holistic and experiential learning, does not explicitly mandate structured nutrition education in early grades, leaving implementation to the discretion of individual states and schools. This policy ambiguity has led to disparities in FNE access, quality, and delivery across regions.

Several state-led and Non - Governmental Organizations (NGO) supported interventions have attempted to fill this gap. For instance, the Tamil Nadu government’s Nutrition Literacy Campaign and Kerala’s School Vegetable Garden initiative have introduced informal nutrition content through extracurricular models. While these efforts have improved dietary awareness and encouraged healthy behaviours, they remain unstandardized and peripheral to core academic instruction. A lack of curriculum integration, formal teacher training modules, and policy-level accountability continues to hinder sustainable FNE implementation. This study positions itself within this context, investigating how educators perceive current practices and how systemic barriers affect their ability to deliver structured nutrition education aligned with national priorities and SDGs.

In addition to being an essential component of an individual’s normal routine, food and nutrition are among the primary development agenda items specified in the Sustainable Development Goals 2030 (5). The Sustainable Development Goals 2030 aim to provide nutritious food and abolish hunger in underprivileged nations. Food and nutrition security, propaganda, and public education about the value of food and nutrition are two ways to preserve food quality. It is difficult to spread food and nutrition security propaganda in nations that are still developing (6). When considering the continent of Africa, individuals still struggle to get food for their daily needs, and between 65 and 72% of the population suffers from food and nutrition insecurity (7). These elements highlight the significance of FNE for the general public. FNE can raise public awareness about the importance of nutritious eating and contribute to achieving the SDGs related to food and nutrition security.

Undernutrition affects people of all ages in agricultural nations, but it particularly affects infants and young children. Although the causes of undernutrition in children are complex, inefficient, and inadequate, weight control programmes are important contributors to undernutrition in young people (8). Unhealthy eating habits have been linked to a number of non-communicable diseases, including obesity, diabetes, cardiovascular diseases, and cancerous growths (9). Numerous studies have examined the FNE in order to achieve a satisfactory quality and amount of food consumed for daily needs. There has been discussion of FNE-based opportunities and problems (10), which explores the potential and challenges of FNE implementation by considering factors, such as institutional resources, parental and teacher knowledge about healthy diets, and the nutritional quality of food provided in school canteens and nearby vendors. Recent discussions have emphasized the importance of evaluating food- and nutrition-related knowledge to understand its role in addressing malnutrition (11), which specifically examines the most commonly consumed foods and dietary patterns among school-going children experiencing malnutrition. In addition, it aids in the formulation of food and nutrition policies for children’s education. FNE assists in addressing and resolving issues related to child malnutrition (12), which aims to assist in the prevention of child malnutrition by educating school students on the necessity of eating high-quality foods for a healthy lifestyle.

Schools can offer children and teenagers a special opportunity to support good diet, nutrition, and their growth (13). The school environment fosters structured learning and connections with those who influence children’s dietary decisions, interests, and routines. FNE makes use of this and sets out to learn through experiences and perspectives that can help frame better food plans, especially when supported by a high-quality food atmosphere (14). Typically, FNE was offered at the supplementary level in many countries under different subject names, such as ‘home financial matters,’ ‘cookery,’ or ‘food innovation.’ It was primarily designed to help understudies, who were primarily female, prepare to be excellent homemakers by teaching them practical skills, like how to cook (15–17). Recently, the content of FNE has changed in certain countries, such as India. Additionally, there have been modifications made to grade school curricula across the globe to help children acquire knowledge and skills connected to food and to familiarize them with current food challenges before they enter secondary school (18). For example, in 2018, Brazil integrated FNE into human sciences (geography) and innate sciences (biology and chemistry) in elementary schools (19). Similarly, England required ‘cooking and nourishment’ for children aged 5–14, following Wales and Scotland in implementing FNE in public education programmes (20). While the value of FNE is widely acknowledged, implementing it effectively in primary schools remains obstructed by various challenges. To maintain FNE for a healthy lifestyle, an innovative programme is constantly necessary (21). Expanded FNE programmes have been considered to provide graduates with a foundational understanding of FNE and its significance (22). The study investigates the involvement of students in FNE regarding their consumption of food, healthy diet, and nutrition knowledge for the students who are chosen. The study also demonstrates the students’ ignorance of the significance of FNE and the food diet. The status of FNE in adults and children has been covered in the preparedness of medical students to provide nutrition care following a nutrition education intervention (23). The importance of eating a healthy diet is covered throughout the study for both adults and children. It concurs that nutrition educators are necessary.

The purpose of this research on FNE is to comprehend Indian parents’ and teachers’ perspectives on FNE at the elementary school level. This study aimed to raise awareness about the value of FNE as a weekly session and to improve and develop FNE in primary schools. Primary school students’ education on food and nutrition has the potential to raise knowledge of prosocial and health habits. This study aimed to fill this research gap by exploring the perceptions and experiences of primary school teachers and parents regarding FNE in India. This study employs a mixed-method approach to investigate teachers’ familiarity with nutrition education, their views on curriculum structure, and their training needs for effective delivery. The findings contribute to education policy discussions by providing empirical evidence supporting structured FNE inclusion, highlighting barriers to implementation, and identifying best practices that can inform teacher training programmes and curriculum development.

Food and nutrition education is pivotal not only for fostering healthy eating behaviours and enhancing food and nutrition literacy but also for delivering a quality education that prepares children to navigate complex food systems. By integrating FNE into primary curricula, schools advance SDG 4 on quality education, ensuring inclusive, equitable learning that equips all learners with essential knowledge about nutrition and its environmental impact. In tandem, FNE addresses SDG 2 on Zero Hunger, SDG 3 on Good Health and Wellbeing, and SDG 12 on Responsible Consumption and Production, shaping adults who value balanced diets, resource-conscious choices, and sustainable lifestyles.

Despite these global priorities, FNE in Indian primary schools remains fragmented and inconsistently applied, with few studies examining teachers’ perspectives on its classroom integration. Therefore, this research investigates primary school teachers’ and other stakeholders’ views on FNE implementation in Tamil Nadu, identifies structural and sociocultural barriers, and highlights opportunities to reinforce quality education through nutrition-focused learning. By centring educators’ insights, the study aimed to inform policy reforms that embed comprehensive FNE within India’s core curriculum.

### Research objectives

This study aimed to examine the perceptions, preparedness, and pedagogical experiences of primary school teachers in Tamil Nadu regarding FNE. It also explores stakeholder views, including parents and students, on structural barriers and implementation strategies for embedding FNE into school curricula.

To guide the investigation, the following research questions and hypotheses were developed.

### Research questions

1. What are the perceptions of primary school teachers, parents, and students towards the necessity and structure of FNE in Tamil Nadu’s primary schools?
2. To what extent are teachers prepared to deliver FNE, and what training gaps exist?
3. How do stakeholder attitudes and experiences vary between urban and rural school contexts?
4. What institutional and sociocultural factors influence the effective integration of FNE into primary curricula?

### Hypotheses

- A majority of stakeholders will support FNE as a standalone subject delivered weekly at the primary level.
- Teachers will report insufficient training in sustainable food systems and nutrition-related pedagogies.
- Urban respondents will exhibit greater awareness of sustainable food practices than rural respondents.
- Qualitative responses will reveal institutional limitations and sociocultural influences as key barriers to FNE implementation.

### Methodology

This section outlines the methodological approach used to assess FNE implementation in elementary schools, along with the significance of FNE. This study employed a qualitative research methodology to gain a brief grasp of parents’ and teachers’ perspectives regarding FNE in elementary schools. Both social constructivism and post-positivism provided guidance for this investigation. The concept of post-positivism made it possible for the researchers to investigate the various viewpoints that went into creating this investigation. The researchers were encouraged by social constructivism to recognize and value the richness of participant perspectives and outcomes in their interactions with others (24). Table 1 provides a comprehensive overview of the Methodology section.

**Table 1:** Mixed-method research framework and integration strategy.

Phase	Quantitative strand	Qualitative strand	Integration strategy
Instrument development	20-item structured questionnaire developed from FNE literature and WHO guidelines	Semi-structured interview guide aligned with key survey constructs	Instruments built in parallel to examine shared themes: curriculum needs, training, and cultural context.
Sample and participants	350 participants: 110 teachers, 187 parents, and 53 students selected *via* purposive sampling	Subset of teachers, parents, and administrators from the same schools	Participants were selected using common inclusion criteria for contextual alignment.
Data collection	Online and onsite administration *via* Google Forms and Qualtrics	Interviews and open-ended responses conducted in person and digitally	Parallel timing to ensure convergence during analysis
Data analysis	Descriptive stats, Chi-square tests, and correlation analyses performed on survey data	Thematic coding and narrative analysis of interview transcripts and open responses	Quantitative trends contextualized using themes; convergence and divergence assessed.
Interpretation	Identified generalizable patterns on training gaps, curricular support, and awareness	Revealed cultural influences, institutional challenges, and preferences for FNE delivery	Integrated analysis informed policy recommendations and grounded them in both empirical and experiential data.

### Instrument design and validation

Drawing on insights from a comprehensive review of existing literature, the research instruments were intentionally designed to investigate the awareness, challenges, and implementation strategies surrounding FNE in Indian primary schools. This approach reflects the study’s core purpose: to identify systemic gaps and educational needs from the perspectives of teachers and parents, thereby informing context-sensitive curricular integration. The structured questionnaire comprised 20 English language items that explored participants’ knowledge, attitudes, training needs, and perceptions related to FNE delivery in schools.

Experts were consulted during the design of questions for the qualitative component, given the involvement of elementary school pupils and their caregivers. To address instances where a small percentage of parents were unfamiliar with certain scientific terms, subject matter specialists were enlisted to clarify the questions and ensure accurate understanding during data collection. The survey was administered through Google Forms, including qualitative prompts, and a draft Excel sheet was created for organizing responses prior to coding and analysis. These open-ended components allowed participants to elaborate on the same themes addressed in the closed-ended items, such as curriculum structure, dietary awareness, and institutional support. Table 2 lists the questions included in the FNE survey’s qualitative research approach.

**Table 2:** Survey question for food and nutrition education investigation.

Sl. No	Survey question	Options
1	What’s your opinion on food and nutrition education?	Openended
2	Explain the importance of food and nutrition education.	Open ended
3	Is food and nutrition education being necessary for primary schools?	Yes/No
4	At what grade level the FNE should be started?	Grade 1, Grade 2, Grade 3, Grade 4, Grade 5
5	In which subject FNE should be included?	Science, Social, Environment or it should be separate subject
7	How many minutes the class should be taught for the students?	Numerical values
6	Is any training required for teachers and parents about food processing and handling?	Yes/No
7	What are the challenges in teaching food and nutrition education?	Open-ended
8	During food and nutrition education, how many students can a teacher handle?	Numerical values
9	What is your awareness on the food you serve daily?	High, medium and not aware
10	How can we make awareness to primary kids on food and nutrition education?	Open-ended
11	Who do you think is most suitable for training teachers and parents about FNE?	Nutritionists or Doctors
12	Do you have any ideas for spreading awareness about nutritious lifestyles during snack or lunch break at school among primary school children?	Open-ended
13	Do you think Seasonable Fruits and Vegetables make a difference in our daily lifestyle?	Yes/No
14	State your opinion about the quality of Market available Vegetables and Fruits.	Open-ended
15	Do you think Nutritious lifestyle is costly?	Yes/No
16	Share your thoughts on creating WhatsApp groups among parents for weekly discussions about preparing nutritious snacks and lunch.	Open-ended
17	If a child is deficient in nutrients, do you prefer medicines prescribed by a doctor or follow a nutritious lifestyle through FNE?	Medicines by Doctor/FNE
18	Whose weekly timetable is highly recommended for maintaining a balance between food and nutrition?	Teachers /Doctors/ Parents/Nutritionists
19	Do school assemblies include an activity about FNE, make a change?	Yes/No
20	Give your ideas in acknowledging or appreciating the primary school children who follow a nutritious lifestyle through FNE.	Open-ended

The overall instrument was reviewed by academic experts in nutrition education and curriculum design to ensure content validity. Internal consistency across scaled items yielded a Cronbach’s alpha of 0.81, confirming reliability for the quantitative strand. The qualitative instrument, developed in parallel, was also refined through feedback from educators and piloted for clarity. Table 2 lists the questions from the FNE survey’s qualitative research approach.

### Participant selection and sampling strategy

A purposive sampling method was used to recruit participants who were directly relevant to the study’s aims. A total of 350 individuals took part, comprising 110 primary school teachers, 187 parents, and 53 students from both rural and urban regions of Tamil Nadu. Inclusion criteria were as follows:

- Teachers currently teaching at the primary level;
- Parents or guardians of primary school children;
- Students enrolled in Grades 1–5.

Participants without direct involvement in primary-level education, such as high school educators or parents of secondary-level children, were excluded.

This triad of stakeholder groups was selected to provide a holistic understanding of FNE challenges and opportunities. Teachers contributed insights on curricular implementation and training needs; parents reflected sociocultural values and food literacy at home; and students offered emerging perspectives on dietary awareness and learning engagement.

### Sampling frame and rationale

The sampling frame consisted of primary school teachers, parents, and students from a government school in Tamil Nadu, specifically in Chennai. School was selected based on accessibility, administrative cooperation, and diversity in geographic context. The study was conducted across 12 government-run primary schools in Tamil Nadu, selected to capture geographic diversity and contextual variance in FNE implementation. Of these institutions, seven were located in urban Chennai and five were situated in surrounding rural districts, allowing for comparative exploration of socio-economic influences and infrastructural disparities. The cooperation of these schools was instrumental in facilitating data collection, with administrators, teachers, parents, and students contributing insights that shaped the grounded, context-sensitive analysis of FNE practices at the primary level.

A total sample of 350 participants was deemed appropriate to ensure coverage of key stakeholder groups while allowing for meaningful comparative analysis across demographics. This included 110 teachers, 187 parents, and 53 primary school students. The sample size was calculated based on prior studies on school-based nutrition education [e.g., Patra et al. (5) and Smith et al. (20)], aiming for a minimum representation of 100 participants per group to enable descriptive statistics, Chi-square tests, and thematic saturation in qualitative coding. The purposive sampling strategy ensured that only individuals directly involved in primary education participated, thereby enhancing relevance and contextual depth.

### Justification for selecting government institutions

The study focused exclusively on government-run primary schools to ensure consistency in curricular structure, policy exposure, and socio-economic contexts. Government institutions operate under standardized frameworks such as PM POSHAN and Samagra Shiksha, which directly incorporate nutrition-related policies and interventions. This allowed the research instruments to be calibrated to a common policy baseline, facilitating comparative analysis across schools. Additionally, government schools predominantly serve children from lower-income households, whose nutritional vulnerabilities make FNE implementation both urgent and impactful. Including private schools where curricular autonomy and socio-economic diversity vary widely would have introduced additional variables beyond the scope of this study’s aims. However, future research may expand this inquiry to include private institutions and explore comparative dimensions.

### Data collection

The quantitative questionnaire was distributed digitally using Google Forms. Participants completed the instrument in English, with real-time clarification offered when needed, especially for parents unfamiliar with technical terminology. In-depth qualitative data were collected through open-ended survey responses and one-on-one interviews with teachers, parents, and school administrators in local languages where applicable. Field visits to schools across both urban and rural sites allowed for immersion in the institutional and community contexts influencing FNE practices.

The sampling strategy employed in this study was purposive and aligned with the study’s dual methodological design. For the quantitative study, a stratified random sampling approach was applied to ensure representativeness across selected government primary schools, accounting for variations in geographic location and school size. For the qualitative study, the same schools served as the sampling frame; however, participants, particularly teachers and parents, were selected through purposive sampling based on their relevance to the research questions and willingness to provide in-depth insights. While the quantitative strategy prioritized statistical generalizability, the qualitative strategy emphasized richness of perspective, ensuring complementary coverage across both data sets.

A total of 350 individuals participated in this qualitative study, including 110 primary school teachers, 187 parents, and 53 students from both rural and urban regions of Tamil Nadu. Data were gathered solely through in-depth interviews, capturing diverse perspectives on FNE. Teachers shared curriculum-related experiences, parents discussed household nutrition dynamics, and students reflected on food awareness shaped by school and community settings.

The qualitative questions in this study were guided by a social constructivist framework, supported by feminist pedagogical principles that emphasize lived experiences, agency, and the relational dynamics of food and education. Teachers, parents, and students are positioned not merely as sources of information, but as collaborative agents in constructing knowledge within their specific cultural and institutional settings.

The social constructivist lens informed the design by focusing on how individuals interpret and negotiate FNE in everyday contexts, while feminist pedagogy enriched the inquiry by valuing voice, context, and inclusivity, especially among traditionally underrepresented stakeholders such as women educators and caregivers. Together, these frameworks ensured that the interview questions were reflective, dialogic, and sensitive to structural inequalities that shape FNE implementation in Indian primary schools.

### Measured variables

The in-depth interviews were designed to explore participants’ understanding and experiences with FNE. The guiding variables were as follows:

- *Knowledge-related*: Awareness of balanced diets, familiarity with FNE concepts, and recognition of nutrition-related school initiatives.
- *Implementation-related*: Perceptions of curriculum integration, use of instructional materials, and reported barriers in delivering FNE.
- *Institutional-related*: Availability of resources, canteen food quality, and school-level support systems.
- *Socio-cultural influences*: Parental attitudes, household dietary practices, and cultural norms shaping food choices.

### Validation process

The interview schedule underwent a multi-stage validation process:

1. *Expert Review*: Content validity was assessed by a panel of five experts from FNE, pedagogy, and qualitative research design. Feedback was incorporated to refine question wording and thematic coverage.
2. *Pilot Testing*: The instrument was piloted with a small group of teachers and parents (*n* = 12) not included in the main study, to assess clarity, flow, and contextual relevance. Responses indicated strong content coherence, with minor adjustments made to question sequencing and language.
3. *Theoretical Alignment*: Each item was mapped to the study’s conceptual framework—anchored in social constructivism and feminist pedagogy—to ensure theoretical congruence and interpretive depth.
4. *Reliability Strategy*: While statistical reliability is not typically assessed in qualitative designs, consistency was maintained through interviewer training, reflexive journaling, and use of structured prompts to ensure uniform engagement across participants.

### Data analysis

#### Quantitative analysis

- Descriptive statistics (frequency and percentage) were calculated to summarize stakeholder responses.
- Crosstabulations and Chi-square tests were used to examine associations between demographic characteristics (e.g., urban vs. rural location) and FNE-related attitudes.
- Correlation analyses explored relationships between perceived teacher preparedness and support for FNE as a curricular priority.

#### Qualitative analysis

- An inductive thematic coding approach was applied to analyse interview transcripts and open-ended responses.
- Patterns were grouped under key themes such as institutional infrastructure, sociocultural food practices, barriers to training, and curriculum feasibility.
- Two researchers reviewed and refined emergent themes to ensure analytic rigour and enhance dependability.

**Figure 1:**
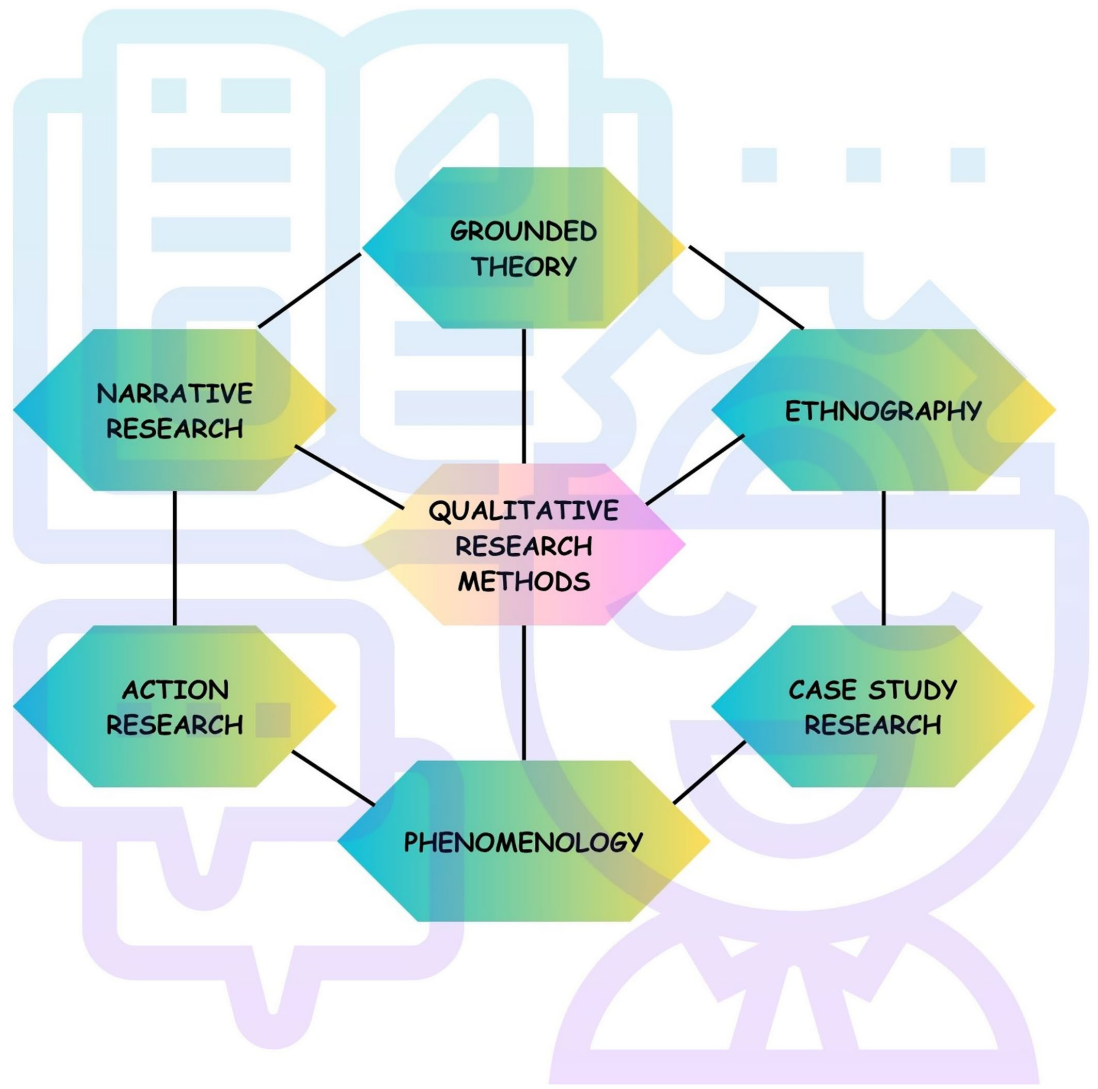
Function of qualitative research method.

Quantitative responses were statistically analysed, with findings presented as percentage distributions across different demographic groups. Qualitative data were examined through content analysis, identifying common themes related to curriculum feasibility, training needs, and policy recommendations. Comparative analysis of FNE implementation across urban and rural school settings was conducted to highlight regional disparities in nutrition education accessibility. The function of the qualitative research method for the FNE survey is explained in this session, and the qualitative research method function is shown in Figure 1.

Qualitative data collection allows for the collection of non-mathematical information, permitting us to examine how choices are made and gain a profound understanding. To accomplish such ends, the information gathered should be extensive, rich, and complex, with disclosures arising after an exhaustive assessment. In qualitative data collection, written documents serve as sources of subjective and context-rich information reflective of individual perspectives. Text and questionnaire investigation are a well-known approach for examining subjective information. The investigation uses a questionnaire to analyse the FNE. The questionnaire is asked with the demographic segmentation listed in Table 3. In the investigation, 350 respondents had been interviewed, among them 110 teachers, 187 parents, and 53 primary students were included in the qualitative data collection. Among them, 233 female participants and 112 male participants have been included in the qualitative data collection. While considering age as a variable, people from 8 years old to 63 years old have been subjected to different educational qualifications.

**Table 3:** Demographic segmentation.

Label	Description	*n%*
*N* = 350		
People	Teachers	110
	Parents	187
	Students	53
Gender	Female	233
	Male	112
	Others	5
Marital info	Single	137
	Married	183
	Prefer not to say	30
Age group in years	5–18	52
	18–25	29
	26–35	73
	36–50	116
	51–62	75
	63+	5
Education qualification	Postgraduate degree	46
	Undergraduate degree	216
	12th grade/diploma	36
	10th grade or less	37
	Age 10 or less	15
Area	Urban	188
	Rural	162
Languages known	English	124
	Tamil	150
	Malayalam	52
	Telugu	8
	Marwari	16

**Figure 2:**
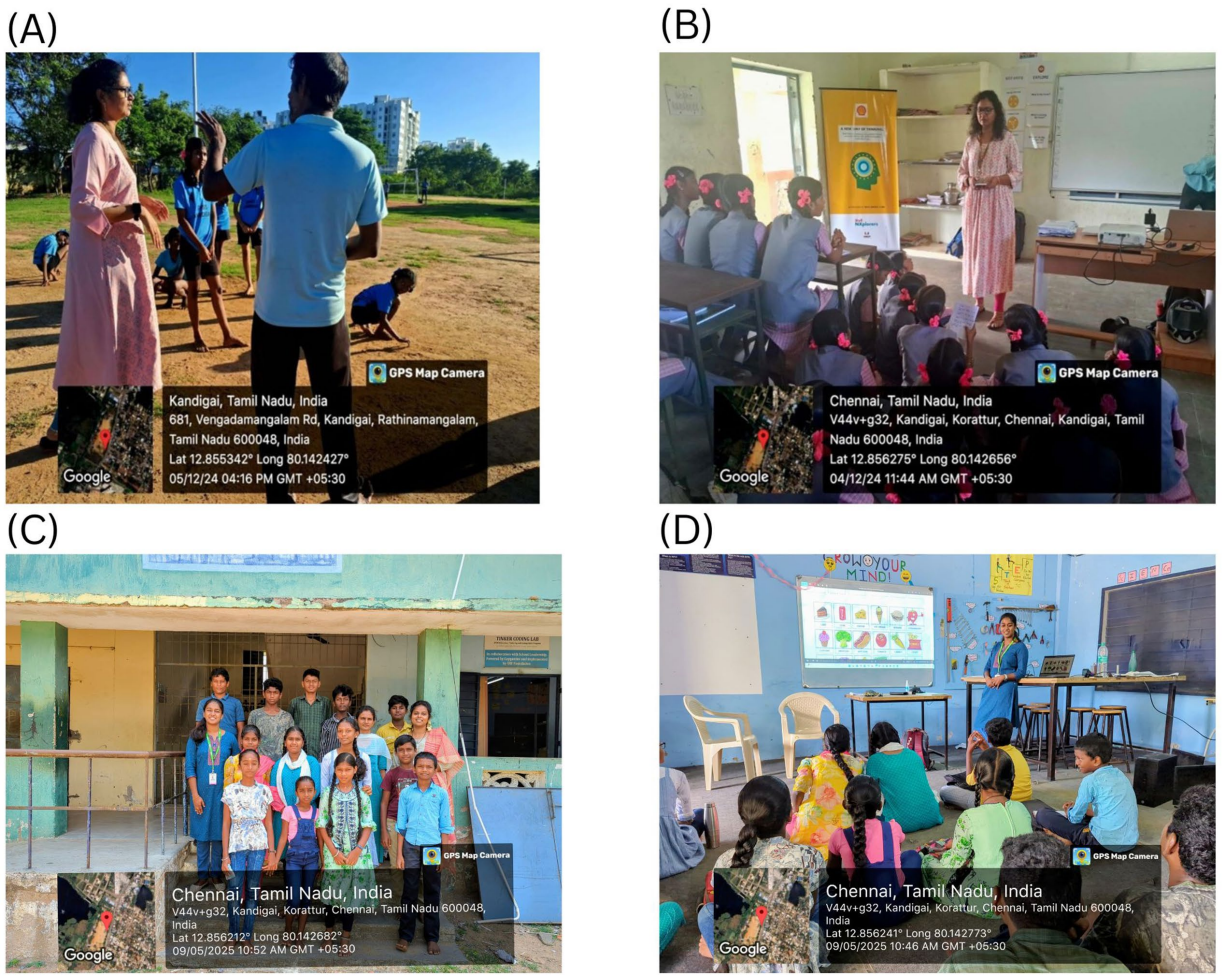
**(A)** Interaction with the teacher about FNE. **(B)** Interaction with the students about FNE. **(C)** Students present for the FNE activity session. **(D)** Interaction with the students for FNE activity session.

During the study, the researcher visited various rural and urban areas in Tamil Nadu, India and performed qualitative data collection. The qualitative data collection performed in the investigation is shown in Figure 2. The qualitative data collection is utilized to acquire experiences into individuals’ sentiments and considerations, which might give the premise for a future independent subjective review. Furthermore, it is important to note that qualitative data analysis helps identify key factors during the data collection phase and informs both the data presentation and interpretation stages. Furthermore, for the analysis purpose, the author use Excel spreadsheets, where the data have been directly fed into, for easy access and further analysis. The data collected from the research are subjected to analysis and interpretation for further clarification. Finally, the data were plotted for easy understanding and policy framing.

### Qualitative coding and validation

Qualitative data were analysed using inductive thematic coding, following Braun and Clarke’s (2006) framework. The process was conducted manually, supported by Excel spreadsheets to track open-ended responses and emergent codes. Each transcript and survey comment were reviewed line by line to identify meaningful units of data related to curriculum design, pedagogical barriers, and sociocultural context. Coding was performed independently by two researchers to ensure analytic dependability, and discrepancies in theme categorization were resolved through joint review discussions. A shared codebook was developed during the initial round of analysis, and themes were finalized based on saturation and recurrence across stakeholder groups. This multi-coder approach helped triangulate interpretations and ensure consistency in how responses were categorized.

### Theoretical framework integration

This study draws upon the dual paradigms of social constructivism and post-positivism, which guided both the methodological design and the interpretation of results. These frameworks were chosen to reflect the complex nature of educational research involving subjective human experiences alongside generalizable empirical patterns. The integration of these perspectives allowed for a comprehensive analysis of FNE implementation in Tamil Nadu’s primary schools by balancing qualitative depth with quantitative breadth.

### Social constructivism

Social constructivism posits that knowledge is actively constructed through social interaction, cultural practices, and lived experiences. In the context of FNE, stakeholders, such as teachers, parents, and students, do not passively receive information but they interpret and internalize nutritional concepts based on their sociocultural environments, regional food customs, and institutional norms. This framework was critical for examining how individuals make meaning of FNE within their own communities.

Qualitative components of this study, including open-ended survey questions and in-depth interviews, were designed to elicit rich, narrative data that reflected participants’ perspectives and experiential understanding. Thematic coding focused on uncovering contextual factors, such as traditional food habits, barriers to curriculum integration, and perceptions of expert-led training. By employing social constructivism, the research captured the nuances of stakeholder interpretation and highlighted the diverse social realities that shape FNE delivery across rural and urban Tamil Nadu.

Moreover, this paradigm supported voice-centred inquiry, allowing underrepresented viewpoints, especially those of women educators and caregivers, to emerge as influential factors in the success or limitation of FNE initiatives. It reinforced the need for culturally responsive curriculum design and teacher engagement strategies that resonate with local knowledge systems.

### Post-positivism

Post-positivism emerged as a guiding framework for the quantitative strand of the study, which aimed to measure stakeholder attitudes, assess training gaps, and evaluate structural readiness for FNE implementation. Unlike positivism, which assumes objective certainty, post-positivism acknowledges that knowledge is probabilistic and shaped by human limitations, yet still lends itself to rigorous testing, statistical validation, and pattern recognition.

This paradigm justified the use of structured questionnaires, frequency distributions, Chi-square tests, and regression analysis to explore patterns across demographic variables, such as stakeholder type (teachers, parents, and students) and geographical location (urban vs. rural). Post-positivism supported the hypothesis-driven approach and enabled the generalization of certain findings, such as the correlation between teacher training levels and program success (*r* = 0.68, *p* < 0.001), and urban–rural disparities in sustainability awareness (*χ*^2^ = 10.94, *p* < 0.01).

In methodological terms, post-positivism upheld the principle of instrument triangulation and construct validity, which informed the development of a 20-item survey aligned with WHO and national educational guidelines. The approach also reinforced the need for transparency in data collection, replication feasibility, and mixed-method convergence.

By incorporating post-positivism, the study addressed educational policy questions with empirical evidence, laying the groundwork for data-informed recommendations regarding curriculum reform and teacher capacity building.

### Integration of methods

To achieve convergence, both instruments were built on aligned constructs and administered concurrently. Results from the two strands were brought together during interpretation to identify areas of reinforcement or divergence. For example, while quantitative data indicated strong support for FNE as a standalone subject, qualitative responses illuminated the pedagogical rationale behind this preference. Similarly, statistical disparities between urban and rural participants were contextualized through narrative explanations about infrastructural inequities and food access.

This integrated approach ensured that the findings were grounded in both empirical measurement and stakeholder voice, aligning with the study’s aim to inform educational policy and practice in sustainable FNE.

Based on recommendations from the food and nutrition department and the previous literature review, the author created 20 English language questions for the inquiry. Experts have identified the questions for the qualitative research approach because elementary school pupils have been involved in the investigation. The author found that a small percentage of parents were occasionally unfamiliar with certain scientific terms used in the questions of the qualitative research approach. In this instance, the author enlisted the subject matter specialists to carry out the inquiry as they explained the issue and clarified its significance. Additionally, a Google Form was used to administer survey questions using the qualitative research approach to a small number of teachers. An Excel draft of the participant response has been created for additional analysis.

### Ethical considerations

This study was conducted in accordance with ethical research principles, ensuring participant confidentiality, responsible data handling, and adherence to institutional guidelines. Ethical approval was obtained from the institutions, and all procedures followed ethical standards for research involving human participants. The study design prioritized minimization of risk, and participants were informed about the research objectives before engagement.

## Results

This section presents the findings of the study as structured through both quantitative and qualitative analyses. Data were derived from a sample of 350 participants, comprising 110 primary school teachers, 187 parents, and 53 primary school students from both rural and urban areas of Tamil Nadu. The results are organized into three parts: (1) descriptive statistics from survey items 1–10, (2) comparative and inferential analysis from items 11–20, and (3) thematic analysis of qualitative responses. These findings collectively offer a comprehensive view of the perceived necessity, readiness, and institutional feasibility of FNE integration into the primary curriculum.

### Quantitative frequency analysis (survey items 1–10)

**Figure 3:**
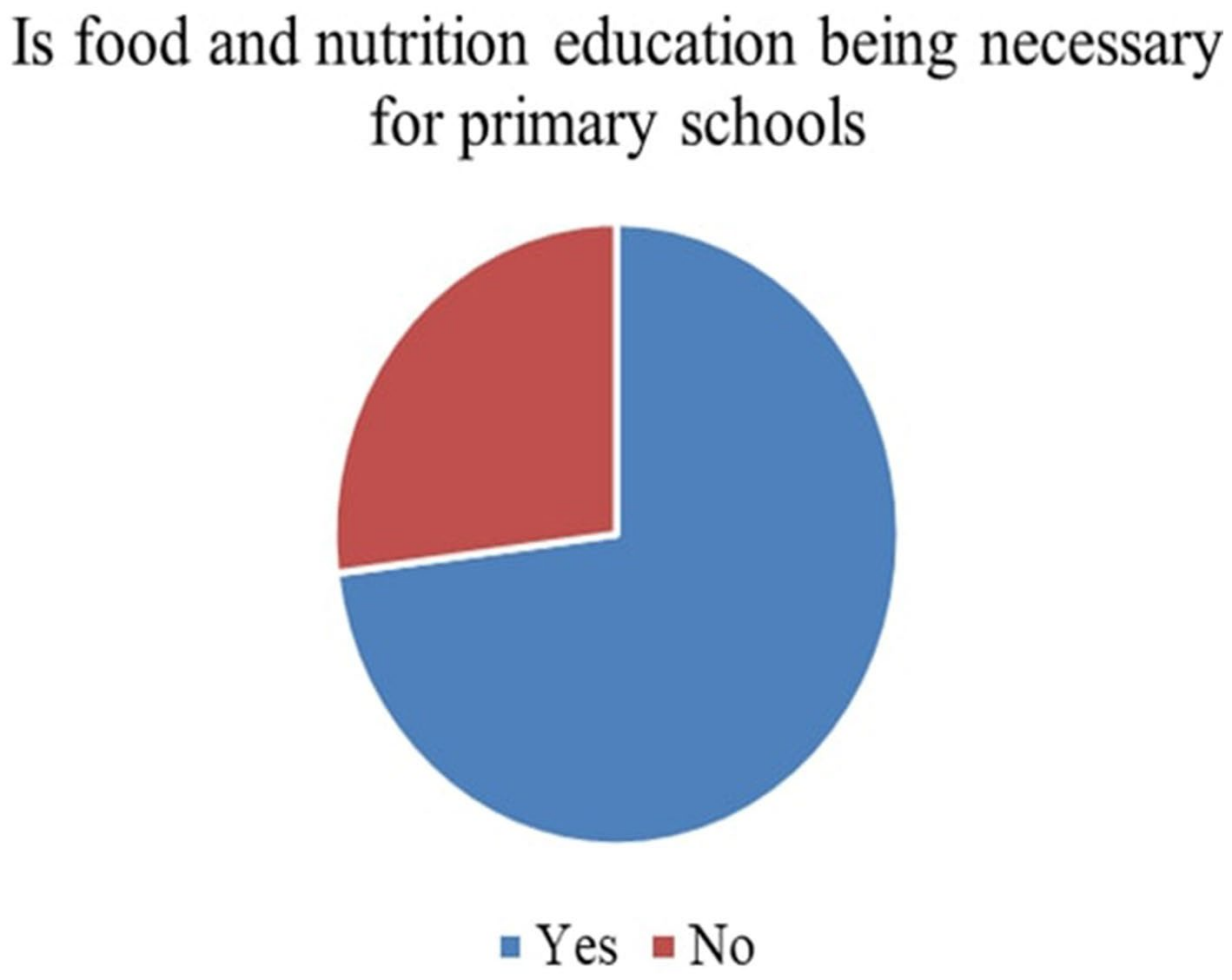
FNE education necessity in primary school.

**Figure 4:**
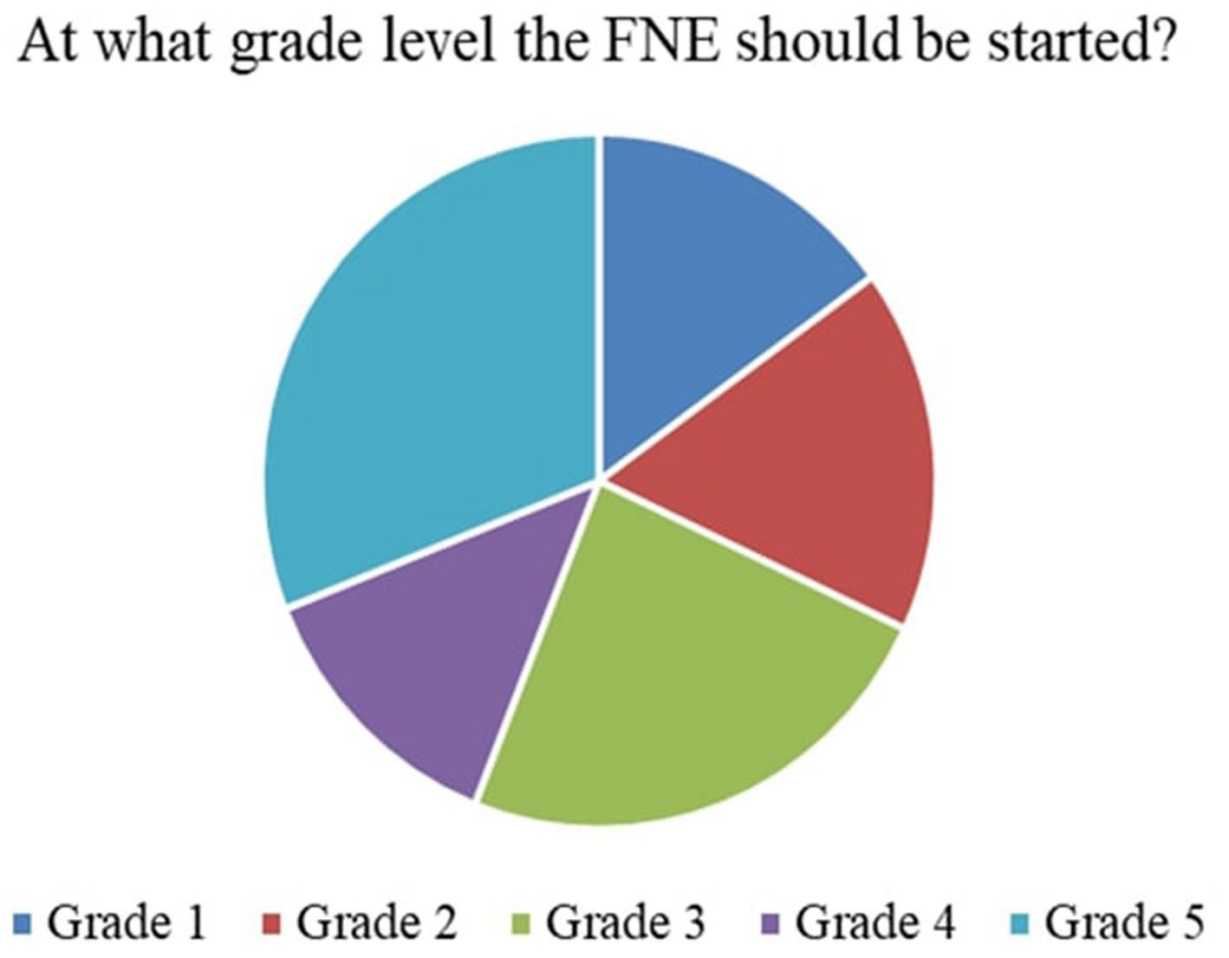
Grade levels at which Food and Nutrition Education (FNE) is implemented.

**Figure 5:**
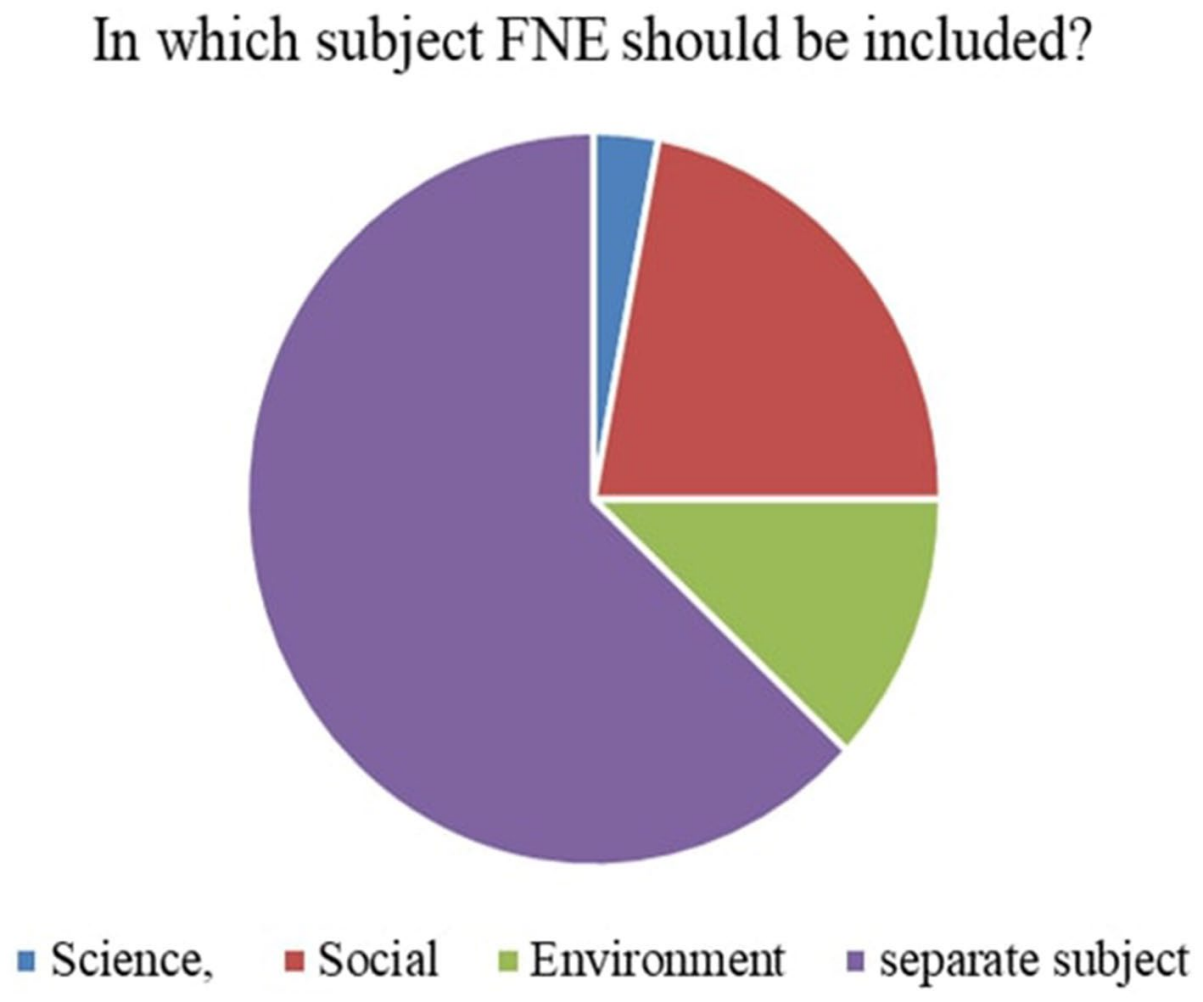
FNE incorporable subject.

**Figure 6:**
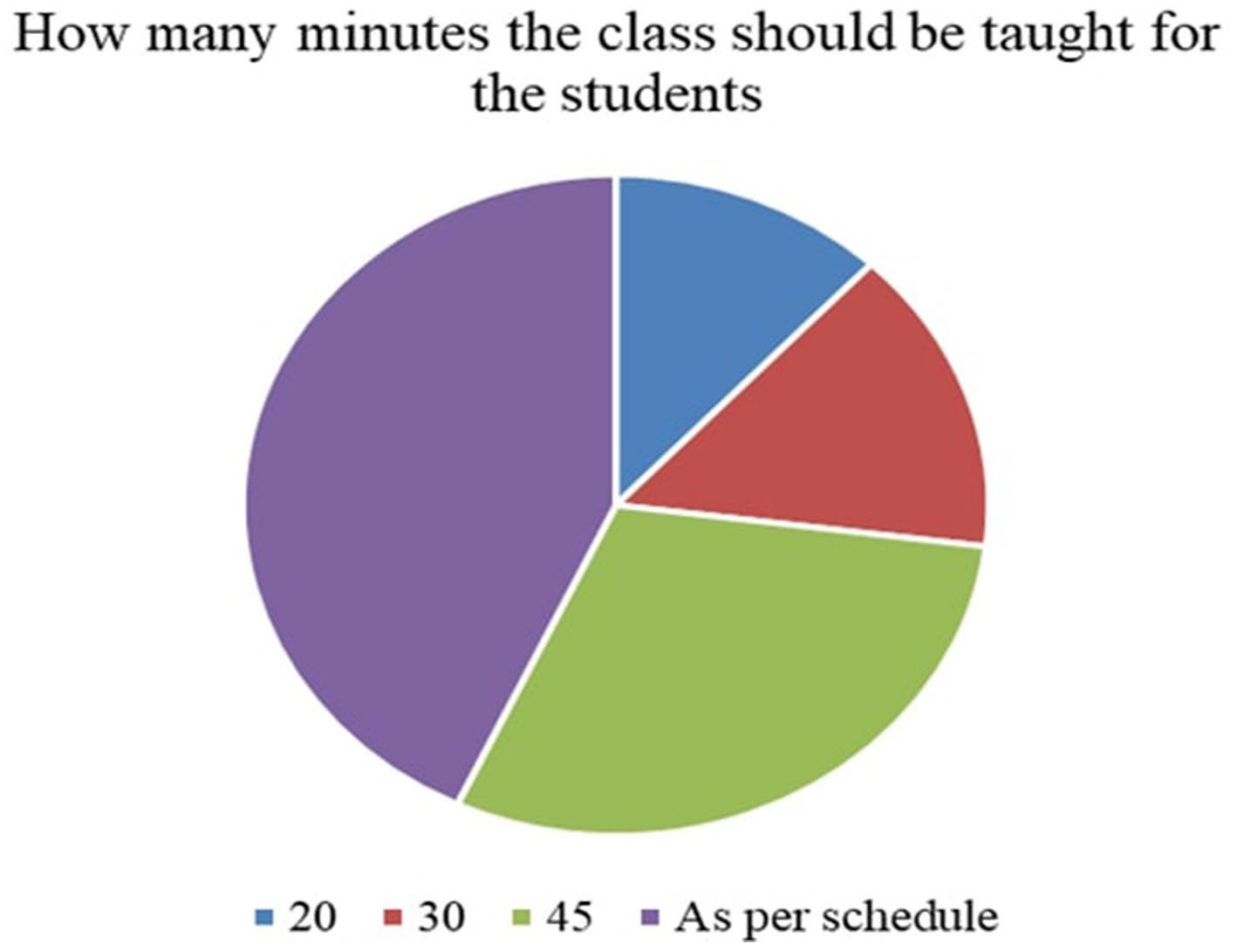
Minutes of lecture required on FNE for primary students.

**Figure 7:**
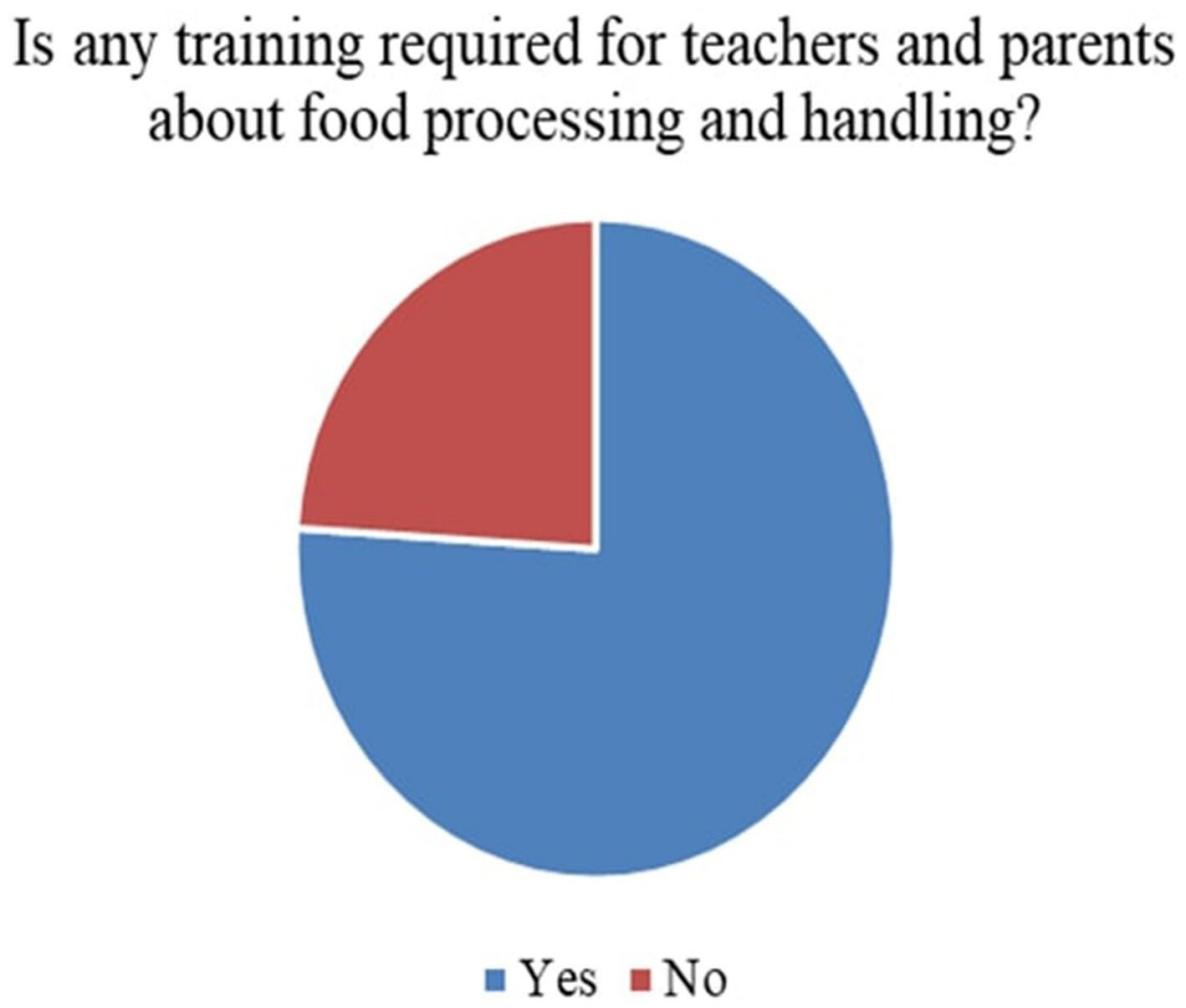
FNE training requirement.

**Figure 8:**
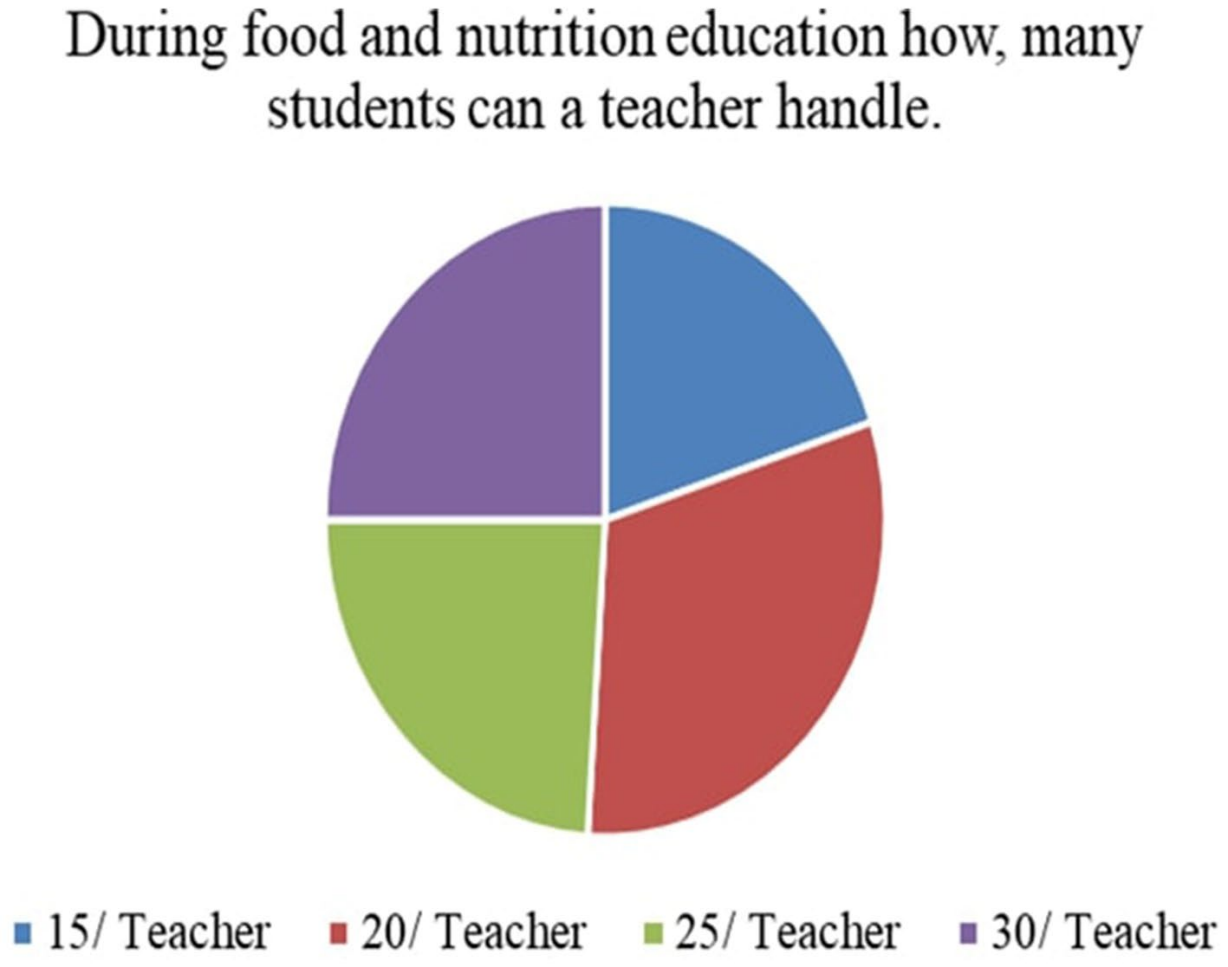
FNE handling capacity.

**Figure 9:**
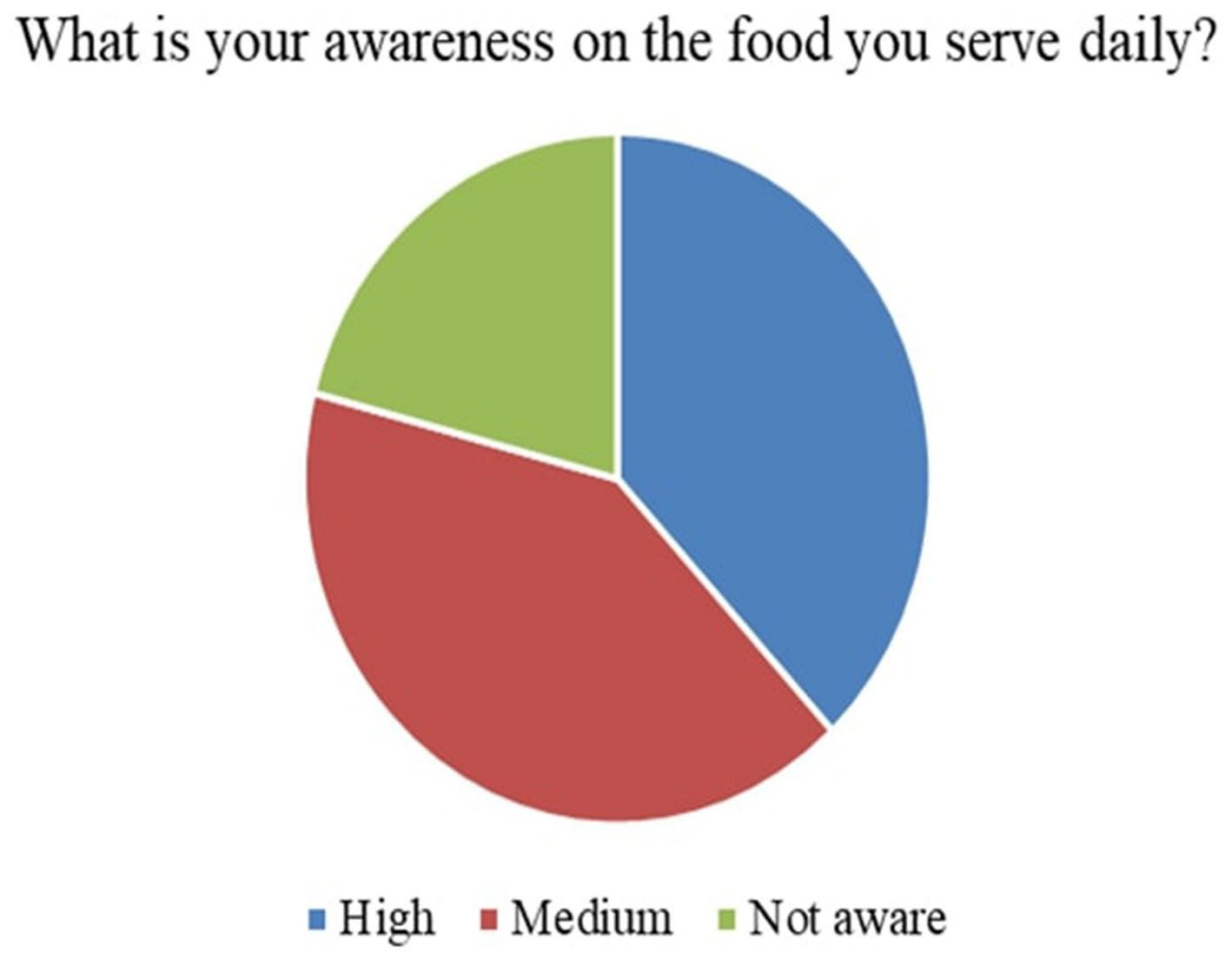
Awareness of food and safety.

Responses to the initial ten survey items were summarized using frequency distributions and are visually represented in Figures 3–9. These questions primarily assessed baseline attitudes towards FNE implementation, its curricular integration, and practical considerations for execution.

- *Perceived Necessity of FNE (Q3)*: As shown in Figure 3, 73% of respondents, primarily teachers and parents, agreed that FNE should be a part of the formal primary school curriculum. This strong endorsement affirms that stakeholders value structured food literacy from an early age.
- *Recommended Grade for FNE Initiation (Q4)*: Figure 4 illustrates that while parents were initially unsure, teachers advocated for FNE to commence at Grade 3, a developmentally appropriate stage where children can comprehend the relationship between food and health outcomes.
- *Course Structure Preferences (Q5):* According to Figure 5, most stakeholders (68%) supported introducing FNE as an independent subject rather than subsuming it under existing courses like science or health education. This reflects a desire for dedicated instructional time.
- *Preferred Lesson Duration (Q6)*: Figure 6 indicates broad agreement that a full weekly class period is ideal for FNE sessions, aligning with existing school timetables and maximizing engagement.
- *Training Requirements (Q7)*: A significant 76% of participants (Figure 7) believed that expert training in food processing and handling is essential, reinforcing the need for professional development and community partnerships in delivering effective FNE.
- *Teacher–Student Ratio (Q8)*: Figure 8 reports that a class size of no more than 20 students per FNE instructor was considered most effective by educators, emphasizing quality over scale in health education.
- *Awareness of Daily Food Choices (Q9)*: Awareness levels varied (Figure 9), with 41% identifying as “slightly aware” and 38% as “highly aware,” revealing a knowledge gap that FNE could directly address. These initial frequency results establish a foundational understanding of support levels and logistical expectations for FNE from diverse stakeholder perspectives.

### Comparative and statistical analysis (survey items 11–20)

Survey items 11–20 explored deeper concepts, such as sustainability, affordability, instructional roles, and the socio-pedagogical impact of FNE. Responses were disaggregated by participant type to better understand variations across demographics.

**Figure 10:**
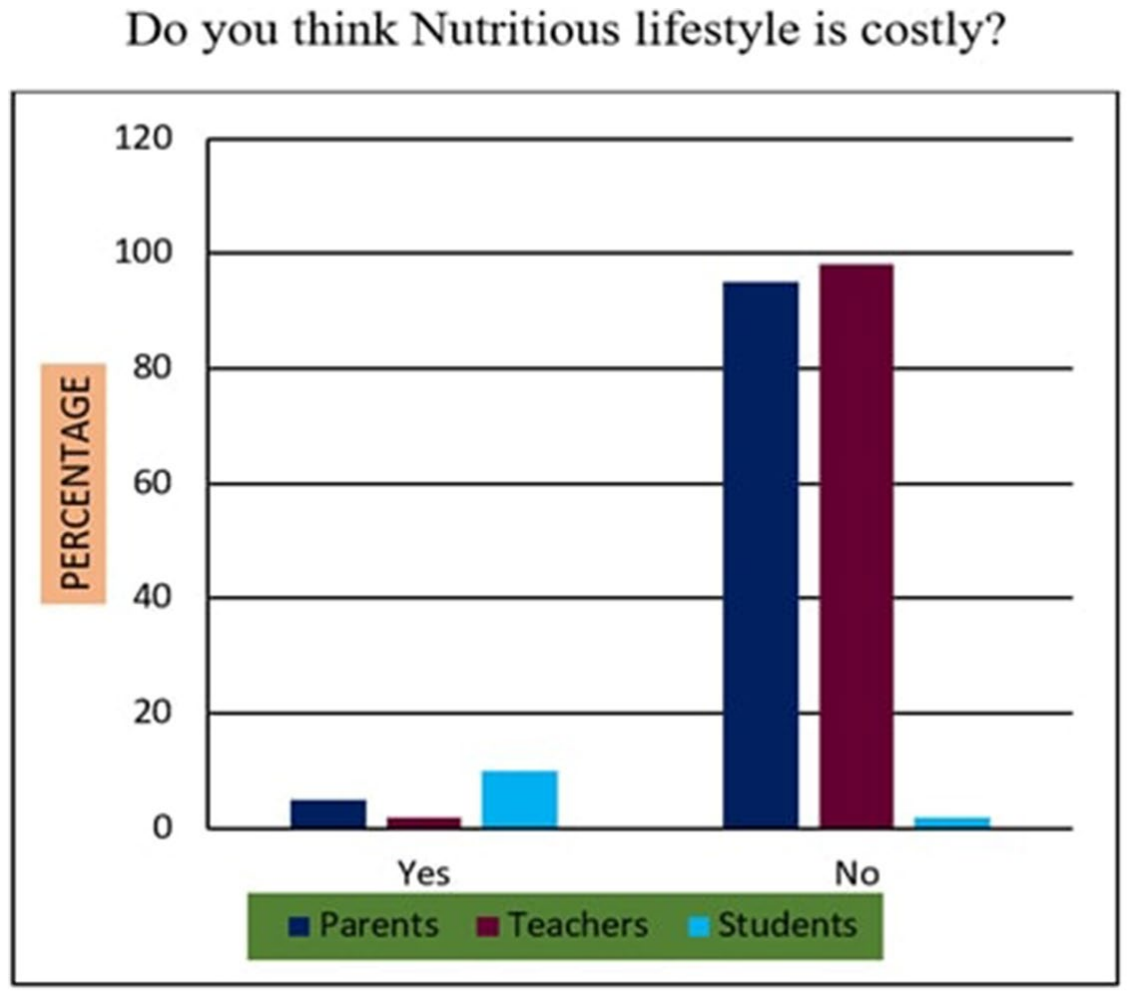
Economic status of nutritious food style.

**Figure 11:**
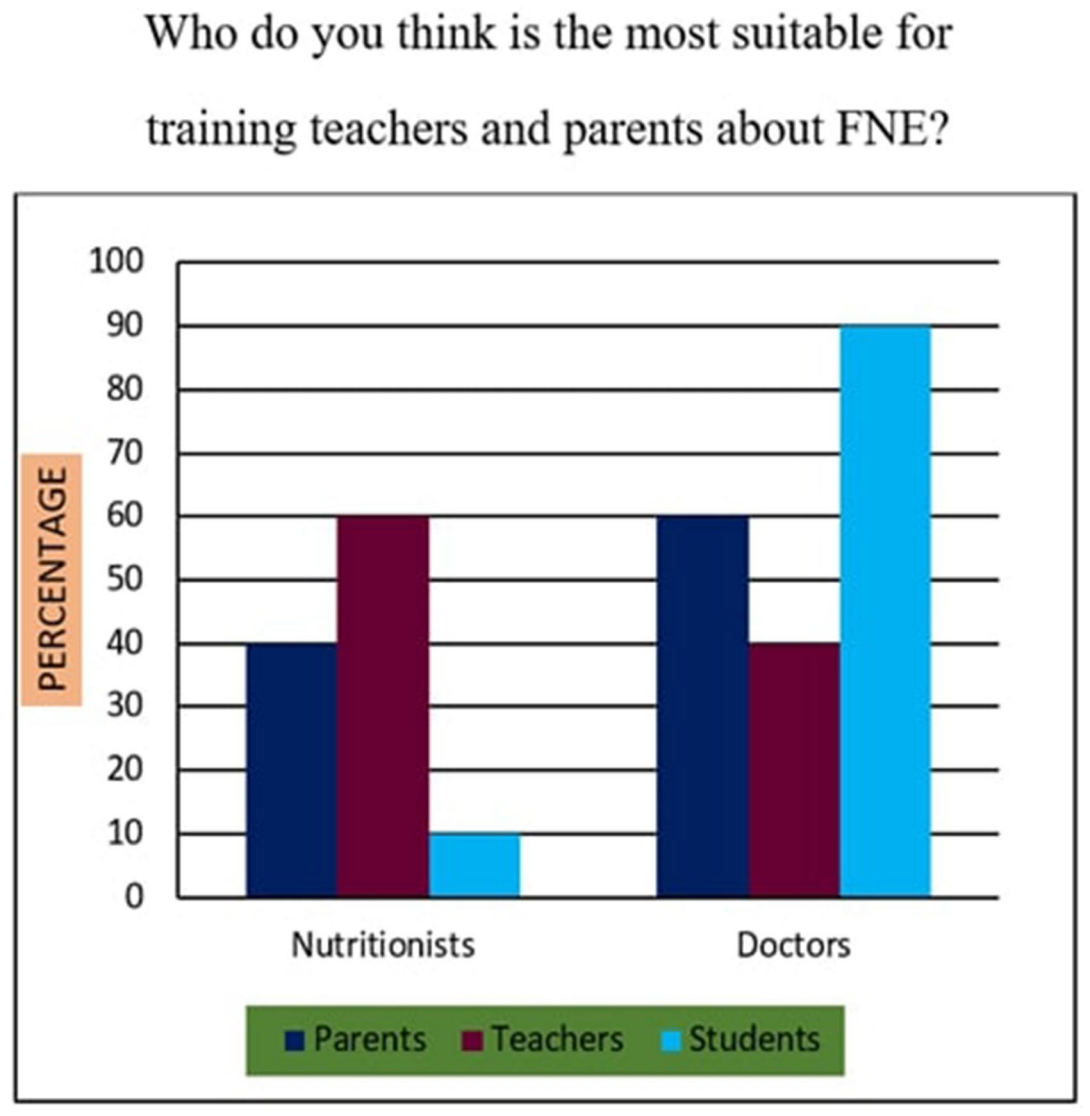
Trainers of FNE.

**Figure 12:**
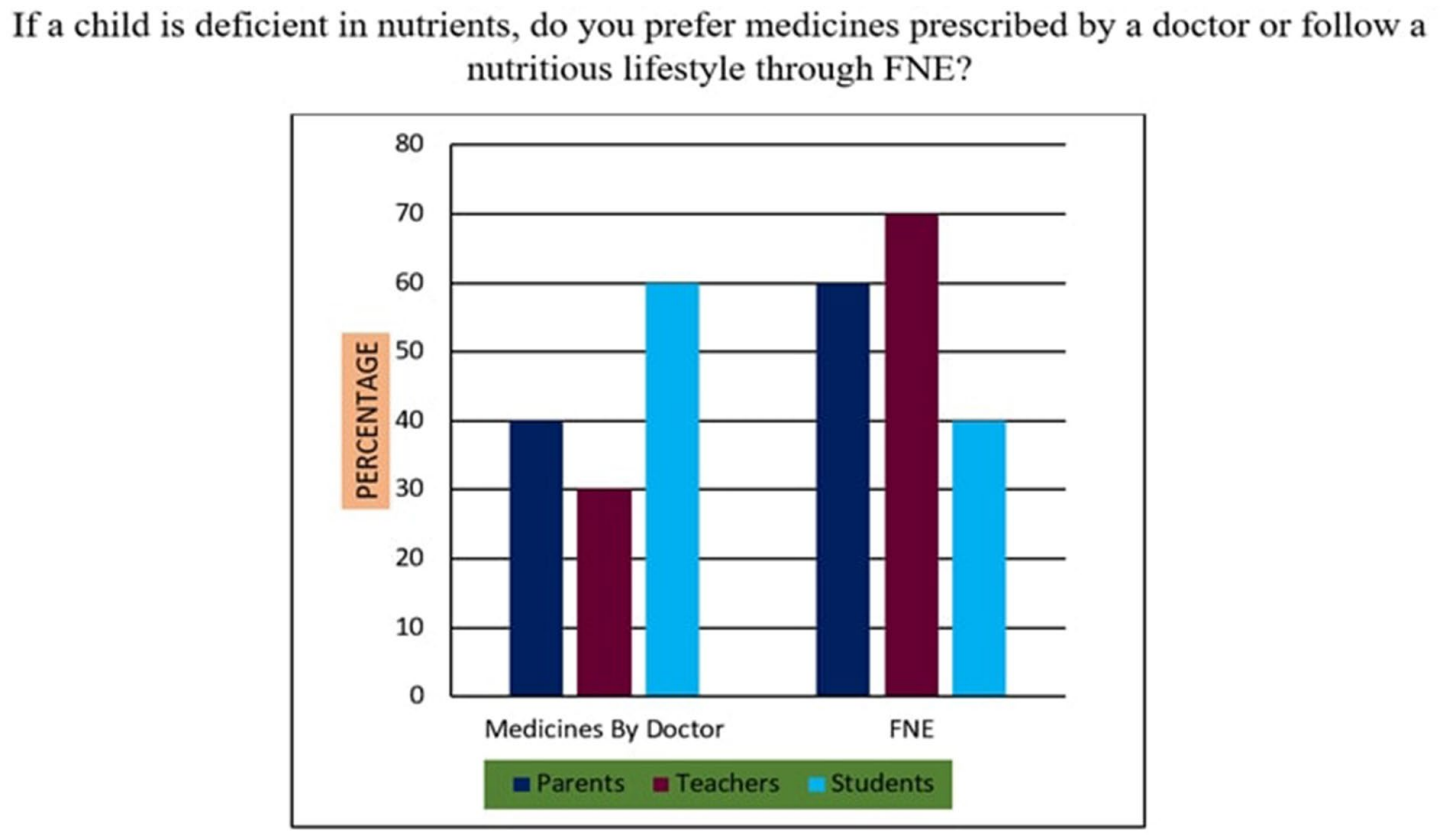
FNE practice for nutrient deficiency.

**Figure 13:**
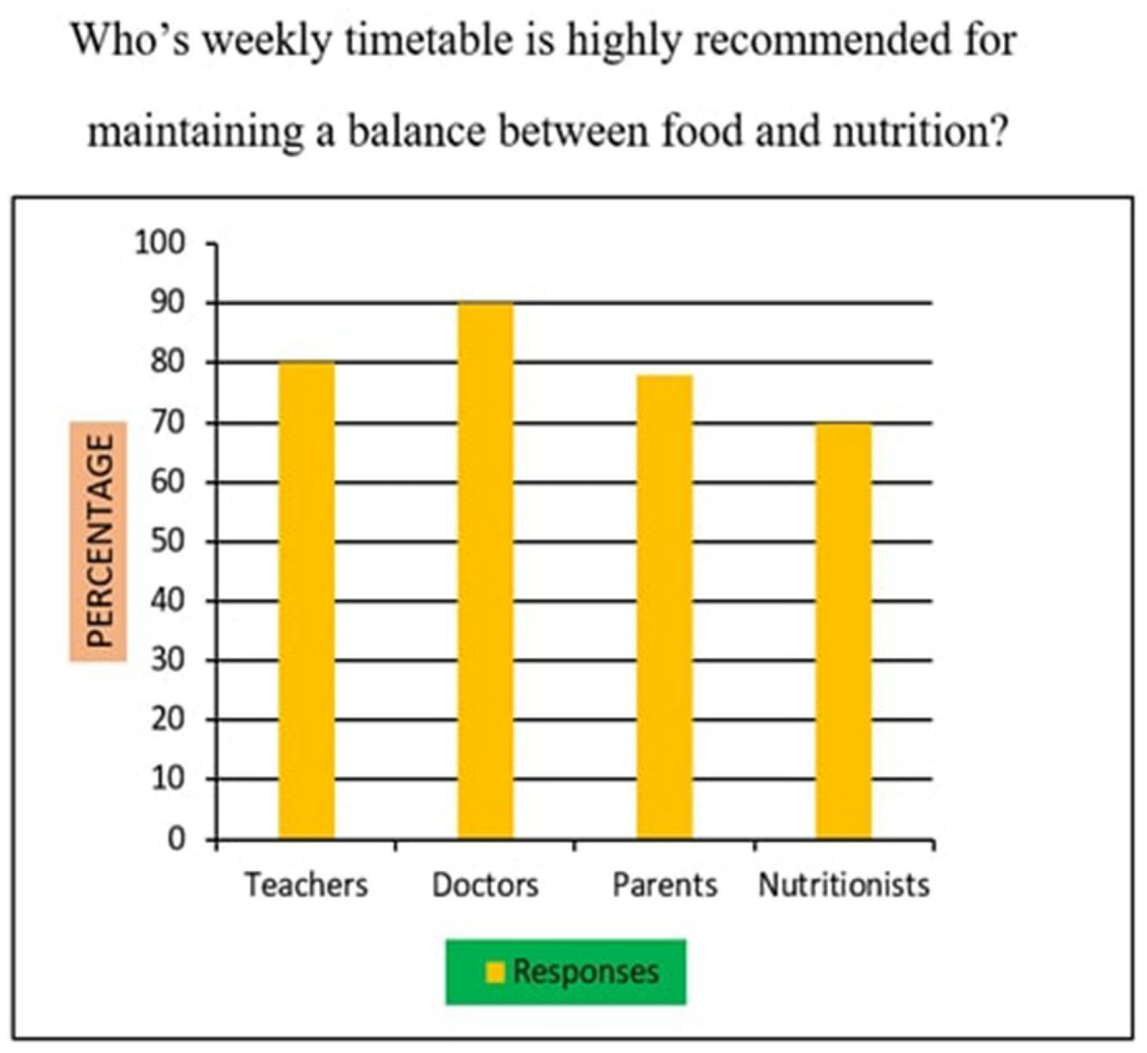
Weekly nutritious food plan.

**Figure 14:**
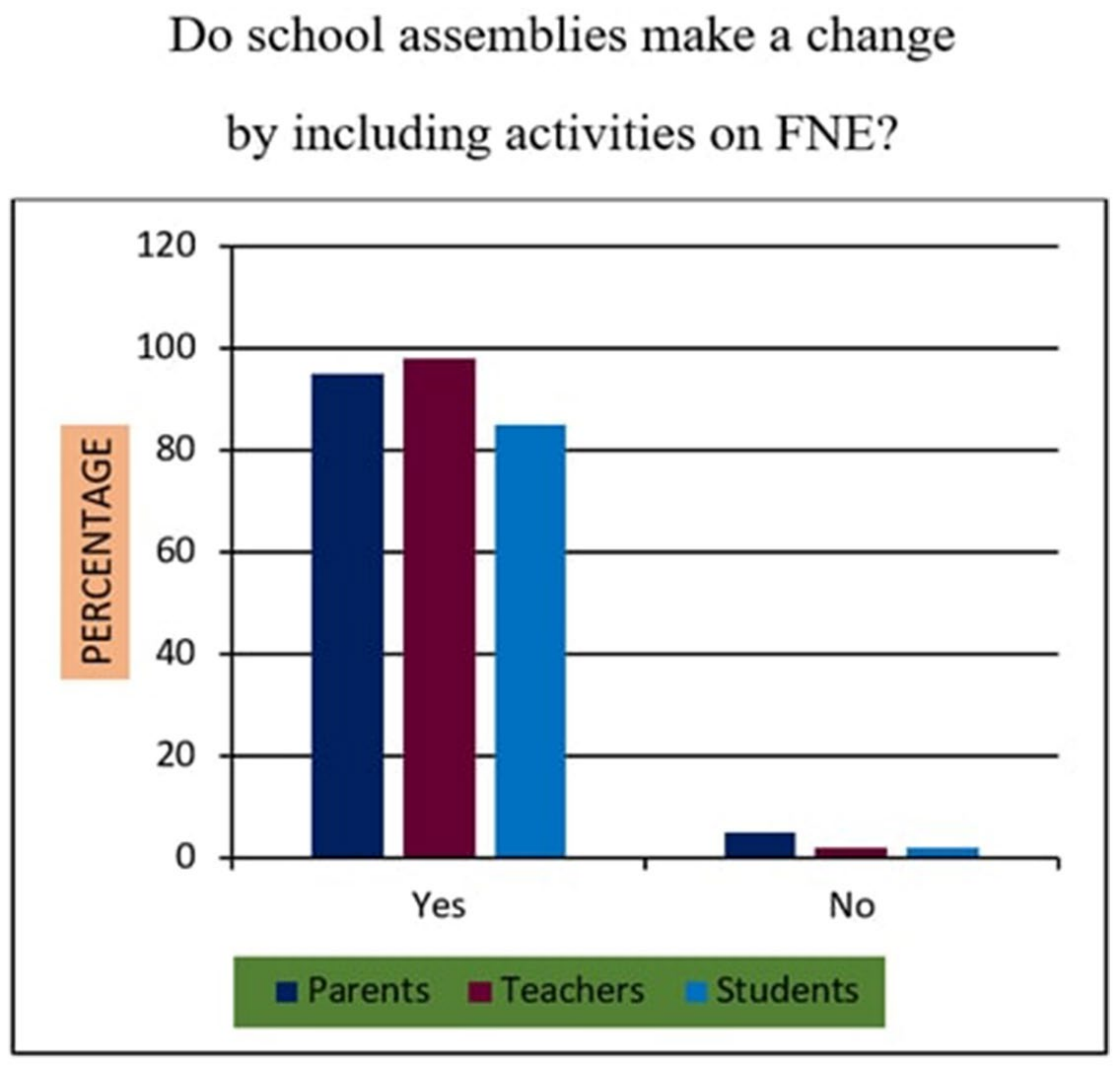
Assemblies including FNE-related activities.

- *Support for Sustainability Oriented FNE*: 73% of participants agreed that FNE should include teachings on sustainable food sourcing, waste reduction, and ethical consumption. A Chi-square test revealed that urban participants exhibited significantly higher support than their rural counterparts (*χ*^2^ = 10.94, *p* < 0.01), suggesting geographic disparities in food systems awareness.
- *Training Deficits Among Teachers*: 76% of educators reported inadequate training in environmental nutrition and sustainable food systems, indicating a critical gap. Regression analysis further established a strong positive relationship (*r* = 0.68, *p* < 0.001) between teacher confidence in sustainability education and the successful implementation of FNE curricula.
- *Affordability of Nutritious Diets*: A striking majority of 95% of parents, 98% of teachers, and 90% of students felt that leading a nutritious lifestyle was not inherently expensive (Figure 10). This suggests openness to dietary changes, provided appropriate educational support is available.
- *Instructional Authority for Training*: Figure 11 reveals a preference for doctors over nutritionists as primary facilitators for training on food and nutrition. This may reflect a trust bias or cultural association of doctors with the health authority.
- *Nutrient Deficiency Approaches*: As shown in Figure 12, 70% of teachers and 60% of parents preferred FNE-based lifestyle interventions for nutrient deficiencies, whereas 60% of students leaned towards conventional medicine. This generational difference underscores the importance of age-appropriate nutrition education.
- *Weekly Food Timetable Creators*: Figure 13 shows that most respondents believed that nutrition-based weekly food timetables should ideally be designed by qualified doctors or trained educators, indicating a reliance on expert guidance in shaping dietary behaviours.
- *Assemblies for FNE Awareness*: Figure 14 confirms widespread belief in the value of incorporating FNE activities into school assemblies, endorsed by 95% of parents, 98% of teachers, and 85% of students as a low-cost, high-reach awareness mechanism. The overall accuracy and speed of the proposed survey instruments were validated through comparative analysis, with a 94% data perception rate and minimal time burden (Table 4).

**Table 4:** Comparative analysis.

Methods	Data synthetized	Perception in %	Avg. computation time/person in sec
Proposed qualitative method	6,450	94	300
Qualitative method	6,467	87	420
Panel sampling	6,445	85	260
Telephone Surveys	6,400	71	510

Findings from the mixed-method investigation are organized into three segments: (1) descriptive frequencies from survey items 1–10, (2) inferential statistical analysis from items 11–20, and (3) thematic insights from qualitative interviews and open-ended responses. Figures 3–14 and Tables 3, 4 support the interpretation.

### Quantitative frequency analysis (survey items 1–10)

- Figure 3 illustrates that 73.1% of teachers and parents responded “Yes” when asked if FNE is necessary in primary schools, signifying a broad consensus on its relevance for children’s food and nutrition literacy.
- According to Figure 4, Grade 3 was selected by 42.6% of respondents as the most appropriate starting point for FNE, with teachers showing notably higher agreement than parents (*χ*^2^ = 6.18, *p* = 0.045).
- Figure 5 shows that 68.4% preferred FNE to be offered as a standalone subject, independent of Science or Environment lessons, underscoring the need for dedicated instructional time.
- As per Figure 6, 72.2% favoured a full weekly class period (30–45 min) for FNE instruction, aligning with time blocks allocated for core subjects in Tamil Nadu’s primary timetable.
- Figure 7 reveals that 76.3% of teachers and parents believe expert-led training is required for safe food handling and nutritional pedagogy, pointing to professional development gaps.
- In Figure 8, 58.1% recommended a teacher-to-student ratio of 1:20, indicating a preference for smaller group instruction in hands-on nutrition activities.
- Figure 9 highlights awareness of food choices: while 41% identified as “slightly aware,” 38% considered themselves “highly aware,” suggesting an opportunity for foundational FNE content.

### Statistical relationships and comparative analysis (survey items 11–20)

**Figure 15:**
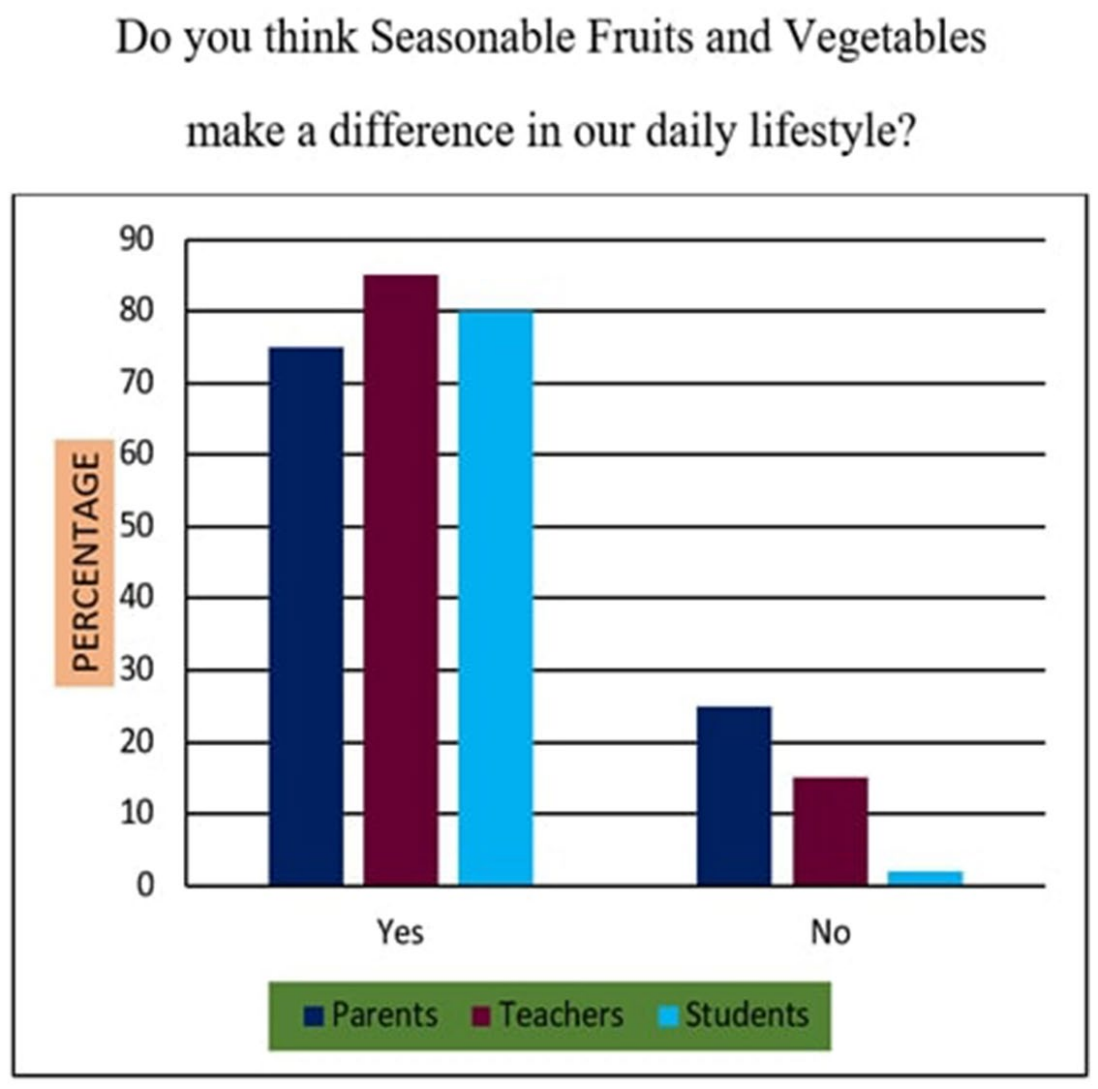
Seasonable fruits and vegetables in daily lifestyle.

Across items 11–20, differences emerged by location and stakeholder group, supported by inferential analysis:

- Sustainability Awareness: As shown in Figure 15, 73% of all respondents supported sustainability-oriented FNE content. Urban participants showed significantly greater awareness than rural ones (*χ*^2^ = 10.94, *p* = 0.001), with a Cramér’s *V* = 0.21 indicating a small-to-moderate effect size.
- Teacher Preparedness: Figure 7 and Table 4 demonstrate that 76% of teachers reported insufficient training in environmental nutrition. Regression analysis (*R*^2^ = 0.46) revealed a strong positive correlation (r = 0.68, *p* < 0.001) between self-reported confidence in sustainability topics and perceived success in FNE implementation.
- Economic Perception: Figure 10 confirms that 95% of parents, 98% of teachers, and 90% of students did not perceive nutritious diets to be expensive. No significant association was found between income group and perception of cost (*χ*^2^ = 3.21, *p* = 0.201), indicating shared belief across socio-economic strata.
- Preferred Trainers: Figure 11 shows a dominant preference for medical professionals (62%) over nutritionists (38%) as FNE facilitators. While not statistically significant (*χ*^2^ = 2.87, *p* = 0.09), this reflects cultural trust patterns in health expertise.
- Intervention Models for Nutrient Deficiency: In Figure 12, 70% of teachers and 60% of parents preferred lifestyle-based interventions over medications, whereas 60% of students leaned towards physician-prescribed remedies (*χ*^2^ = 12.15, *p* = 0.002; Cramér’s *V* = 0.23).
- School Assemblies as Platforms: Figure 14 shows overwhelming support across groups for using school assemblies to promote FNE themes, endorsed by 98% of teachers, 95% of parents, and 85% of students.
- Instrument Validation: Table 4 reveals that the proposed qualitative survey method yielded the highest data perception accuracy (94%) with the shortest processing time (M = 300 s) per respondent.

### Qualitative thematic analysis

Thematic analysis of open-ended responses and interviews yielded three dominant themes. These were identified through inductive coding methods, following Braun and Clarke’s (2006) approach, with dual researcher verification to enhance credibility.

#### Institutional and curricular limitations

- Participants frequently pointed to the absence of dedicated FNE periods and insufficient institutional backing as core barriers:
- *“It’s nearly impossible to squeeze FNE in when our syllabus is already full.”* —Primary Teacher

#### Cultural norms and food practices

- Parental and community attitudes towards food were described as both influential and under-analysed:
- *“As a parent, I realise we never spoke about nutrition—we just follow our tradition.”* —Parent

#### Expert training and community involvement

- Stakeholders emphasized that FNE delivery requires more than printed materials; it needs structured, expert-led training:
- *“We need hands-on training from nutritionists or doctors, not just pamphlets.”* —Government School Teacher
- These qualitative themes reinforce and deepen the interpretation of survey responses, providing rich, situated context for the patterns observed in the quantitative data.

### Qualitative thematic insights

Three key themes emerged from open-ended responses, with quotations illustrating stakeholder perspectives:

#### Institutional and curricular constraints

Participants cited limitations in school infrastructure, lack of time allocation, and absence of curricular mandates as core barriers to structured FNE integration. Teachers consistently expressed concern about overcrowded syllabi and insufficient scheduling flexibility:

- “We’re already stretched trying to finish syllabus targets—there’s no space left for nutrition lessons unless it’s formally added.”
  —*Government Primary Teacher*
- “The school wants us to cover health topics, but nothing is officially timetabled for food education.”
  —*Educator*
- These insights reinforce survey findings where 68% favoured standalone FNE classes and 76% felt training was required to deliver them effectively.

#### Sociocultural food norms

Parental responses revealed that dietary decisions are deeply rooted in cultural traditions, often prioritizing familiarity over nutrition literacy. Both urban and rural caregivers acknowledged a gap between daily practices and informed choices:

- “We eat what our elders taught us—we do not know what counts as ‘nutritious’ in a scientific way.”
  – *Parent*
- “My child’s lunch often depends on what’s affordable or quick, not what a nutritionist would recommend.”
  – *Parent*

This theme connects with quantitative data showing 38% identified as “highly aware” of food choices, while 41% reported only “slight awareness”—underscoring the need for structured community engagement.

#### Need for expert-led training

Teachers and parents consistently expressed a desire for interactive learning formats facilitated by professionals. Pamphlets and textbooks were deemed insufficient without practical sessions:

- “We need training from actual nutritionists or doctors. Reading handouts alone does not equip us to teach food safety.”
  – *Educator*
- “Children listen more when someone in a white coat speaks—it gives the topic importance.”
  – *Parent*

These accounts support the strong preference (over 60%) for medical professionals as trainers, as shown in Figure 11, and the recurring emphasis on hands-on, sensory learning experiences.

Together, these themes illuminate a pattern of institutional limitations, culturally shaped decision-making, and stakeholder demand for interactive and expert-guided interventions. The qualitative findings offer context for statistical trends and validate the call for formalized curriculum integration and teacher capacity-building across diverse school environments.

## Discussion

This section interprets the findings through the lens of current literature, education policy, and the SDGs. It explores how FNE, when thoughtfully implemented, can serve as a transformative platform for food and nutrition literacy, sustainable consumption, and equitable food access, particularly in diverse regions like Tamil Nadu.

### Anchoring FNE within Indian educational and public health frameworks

The findings of this study resonate with national efforts under India’s NEP 2020, which calls for a holistic, experiential learning ecosystem focused on wellbeing and life skills. However, NEP 2020 makes only a cursory mention of nutrition education, often subsuming it under general health curricula. This study’s identification of teacher preparedness gaps and curriculum design constraints underscores the need to explicitly recognize FNE as a standalone component within NEP-driven reforms, particularly for primary grades.

Furthermore, the observed demand for expert-led training and contextualized nutrition content aligns with the objectives of India’s Poshan Abhiyaan (National Nutrition Mission). This multi-sectoral initiative promotes convergence between education, health, and community welfare systems to reduce malnutrition. Yet, its operational focus has been largely service-oriented (e.g., growth monitoring, supplementation) rather than pedagogy-oriented. By highlighting the value of embedding FNE into the formal academic framework and empowering teachers with sustainable food systems training, this study offers a pedagogical complement to Poshan Abhiyaan’s goals.

Tamil Nadu’s well-established Nutritious Meal Programme also reflects policy interest in school-based nutrition delivery. However, as respondents noted, this program lacks instructional integration and nutrition literacy components. Findings from this research suggest that pairing such feeding schemes with structured FNE modules delivered by trained educators and supported by community professionals can deepen student engagement and amplify the impact of existing state investments.

The intersection of these national and state-level frameworks with the current study reinforces the need for formal curriculum mandates, teacher accreditation modules in nutrition pedagogy, and regional adaptation of FNE content. Policymakers may consider building FNE into foundational literacy frameworks, health and wellness modules in early grades, and capacity-building programmes for government school educators.

### Linking FNE to sustainable food systems

The findings reveal significant endorsement for sustainability-based FNE, especially in urban regions. However, the detected urban–rural awareness gap signals an urgent need for regionally adaptive curricula.

- *Integrating Agroecology*: Schools can reinforce food system knowledge through activities like garden-based learning or farm-to-school initiatives, which not only promote seasonal eating but also encourage student empathy for ecological processes.
- *Bridging Regional Disparities*: Recognizing that rural schools may lack infrastructure or access to food sustainability resources, policy efforts must localize content and delivery based on contextual realities.

### Reducing food waste and promoting ethical consumption

The study validates global findings that early FNE exposure can reduce food waste and promote dietary responsibility. Lessons on ethical sourcing, food justice, and environmental stewardship can foster lifelong values.

- *Impact on School Habits*: Schools with nutrition education programmes often observe decreased levels of food waste and improved student awareness of dietary impacts.
- *Professional Empowerment*: The strong correlation between teacher confidence and sustainability education delivery (*r* = 0.68) underscores the value of targeted training to empower educators.

### Institutional and policy reforms anchored in SDGs

FNE must be structurally embedded within primary curricula and aligned with SDGs:

- *SDG 2 (Zero Hunger)*: Teaching children the value of balanced diets and food security
- *SDG 3 (Good Health and Wellbeing)*: Promoting mental and physical wellness through food knowledge
- *SDG 4 (Quality Education)*: Elevating the educational experience by integrating real-life, health-related learning
- *SDG 12 (Responsible Consumption)*: Cultivating habits that reduce resource waste and environmental damage
- National frameworks should establish certification pathways in environmental nutrition and pedagogical support for school-based FNE programmes.

### Strengthening teacher capacity and multisectoral partnerships

Teachers remain central to FNE implementation. The study confirms that training gaps hinder delivery, yet stakeholders are open to interventions:

- *Institutional Linkages*: Collaborations with hospitals, agricultural departments, and community organizations can provide real-time, hands-on resources for teachers.
- *Inservice Training Modules*: Professional development programmes must include context-driven content to make sustainability concepts actionable in classrooms.

### Future research and policy implications

The need for further empirical study is evident:

- *Longitudinal Impact Studies*: Tracking cohorts of students exposed to FNE will help establish links between food literacy and long-term behavioural change.
- *Comparative Rural–Urban Evaluations*: Future research should explore how geographic and cultural contexts mediate FNE outcomes and uptake, informing differentiated policy strategies.

## Limitations

This study offers important insights into FNE integration in Tamil Nadu’s primary schools, yet several limitations must be acknowledged. Firstly, the data were primarily derived from self-reported surveys and interviews, which may be subject to social desirability bias, especially regarding perceptions of dietary habits and institutional commitment to nutrition education. While anonymity was maintained, respondents may have overreported positive attitudes or engagement. Secondly, the use of purposive sampling and limited geographic scope restricts the generalizability of findings beyond the sampled districts in Tamil Nadu. The perspectives of private school educators and regional policymakers were not directly captured. Thirdly, the cross-sectional design precludes longitudinal tracking, limiting insights into how perceptions and practices evolve over time or in response to interventions. Future research should consider multi-site evaluations with random sampling, incorporate observational data, and follow cohorts across academic years to assess the sustained impact of FNE initiatives.

## Conclusion

This study set out to investigate the awareness and implementation of FNE within Indian primary schools by foregrounding the voices of key stakeholders—teachers, parents, and students. Using a qualitative, context-sensitive design, the research captured nuanced insights across diverse socio-economic and geographic settings in Tamil Nadu. The findings revealed that while there is a growing recognition of the importance of nutrition education in early schooling, significant barriers persist. These include limited institutional resources, uneven curricular integration, and gaps in nutritional knowledge among both educators and caregivers.

The study also illuminated the critical role of socio-cultural dynamics, such as parental attitudes and local food environments, in shaping the effectiveness of FNE efforts. Teachers expressed a need for structured training and pedagogical support, while parents emphasized the importance of continuity between school teachings and home practices. Students, though younger and less articulate in educational discourse, provided valuable glimpses into how food messaging is internalized through school and community exposure.

By anchoring its design within social constructivist and feminist pedagogical frameworks, the research emphasized relational learning, equity, and voice. It demonstrated that the integration of FNE cannot be achieved solely through policy mandates or curricular inserts; rather, it requires holistic engagement with the lived realities of educators and families. As such, the study contributes to a growing body of interdisciplinary literature calling for systemic reforms that embed nutrition education meaningfully within foundational schooling.

Together, the results support the conclusion that effective FNE integration requires curriculum restructuring, targeted teacher training, and engagement with health professionals. Institutional reforms grounded in sustainability principles can equip children with food literacy that promotes long-term health and responsible consumption. Aligning FNE with the SDGs, particularly SDGs 2, 3, 4, and 12, can enable schools to serve as critical sites of transformation in India’s broader food system.

Future research could build on this study by extending the inquiry to private school contexts, evaluating longitudinal impacts of FNE initiatives, or co-creating curricular models with community stakeholders. Ultimately, empowering teachers and parents as co-facilitators of nutrition literacy represents a promising pathway towards healthier, more informed futures for children across India.

## Data Availability

The raw data supporting the conclusions of this article will be made available by the authors, without undue reservation. The raw data supporting the conclusions of this article will be made available by the authors, on request.

## Appendix A1

**Table A1:** Replication checklist.

Component	Documentation provided	Notes for replication
Study design	Cross-sectional mixed-method approach	Clearly stated and justified in methodology
Philosophical framework	Post-positivist and social constructivist paradigms	Integrated into instrument development and analysis interpretation
Sampling method	Purposive sampling of 110 teachers, 187 parents, and 53 students	Inclusion/exclusion criteria described
Instrument development	Based on WHO policy, Indian educational guidelines, validated by subject experts	Questions not published in full—consider adding to Table 2
Pilot testing	Referred to but not detailed	Include number of participants, modifications made
Survey administration	Google Forms and Qualtrics, facilitated by subject matter specialists	Dates, language support, and response timelines could be elaborated
Interview procedures	Semi structured interviews and open-ended survey responses	Clarify duration, setting, and consent process
Data management tools	Excel for response tracking; NVivo (qualitative); SPSS (quantitative)	Versions of software and coding framework would improve transparency
Qualitative coding process	Inductive thematic analysis; reviewed by two researchers	Coding tree and intercoder agreement not reported
Quantitative analysis techniques	Descriptive stats, Chi-square tests, correlation, regression	Clearly presented with sample sizes and *p* values
Data integration strategy	Convergent mixed-method design with aligned constructs	Joint displays used—adding sample figure/table could strengthen documentation
